# Patterns of Tobacco Use Across Rural, Urban, and Urban-Slum Populations in a North Indian Community

**DOI:** 10.4103/0970-0218.66877

**Published:** 2010-04

**Authors:** Vivek Gupta, Kapil Yadav, K Anand

**Affiliations:** Centre for Community Medicine, All India Institute of Medical Sciences, New Delhi

**Keywords:** Tobacco, urban, rural, peri-urban, bidi (hand-manufactured cigarette)

## Abstract

**Background:**

Tobacco is the leading cause of mortality globally and in India. The magnitude and the pattern of tobacco consumption are likely to be influenced by the geographical setting and with rapid urbanization in India there is a need to study this differential pattern.

**Aim:**

The aim was to study the rural, urban, and urban-slum differences in patterns of tobacco use.

**Settings:**

The study was conducted in Ballabgarh block, Faridabad district, Haryana, and was a community-based cross-sectional study.

**Materials and Methods:**

The study was conducted in years 2003-2004 using the WHO STEPS approach with 7891 participants, approximately equal number of males and females, selected using multistage sampling from urban, urban-slum, and rural strata.

**Statistical Analysis:**

The analysis was done using the SPSS 12.0 statistical package (SPSS Inc., Chicago, IL, USA). Direct standardization to the WHO world standard population was done to and chi-square and ANOVA tests were used for comparison across three study settings.

**Results:**

Self-reported tobacco use among males was as follows: urban 35.2%; urban-slums 48.3%; and rural 52.6% (*P* value <0.05). Self-reported tobacco use among females was as follows: Urban 3.5%; urban-slums 11.9%; and rural 17.7% (*P* value <0.05). More males reported daily bidi (tobacco wrapped in temburini leaf) smoking (urban 17.8%, urban-slums 36.7%, rural 44.6%) than cigarette use (urban 9.6%, urban-slums 6.3%, rural 2.9%). Females using smoked tobacco were almost exclusively using bidis (urban 1.7%, 7.9%, 11% in rural). Daily chewed tobacco use had urban, urban-slum, and rural gradients of 12%, 10.5%, and 6.8% in males respectively. Its use was low in females.

**Conclusion:**

The antitobacco policies of India need to focus on bidis in antitobacco campaigns. The program activities must find ways to reach the rural and urban-slum populations.

## Introduction

Irrefutable evidence has accumulated in the last century on the deleterious effect tobacco use has on the human health. The impact has been studied both in terms of morbidity and mortality. India is now believed to have a high burden of tobacco and its related morbidity and mortality. It has been estimated that among all the people who smoke worldwide, 16.6% live in India, an absolute figure of 182 million.(1) It has also been estimated that in the SEAR-D (the high-mortality developing region of Southeast Asia dominated by India in terms of population), about 18% of all deaths among adult men and 3% of all deaths among adult women were attributable to tobacco, in year 2000.(2) Strong evidence is available in India from large-scale studies on the association between tobacco use and mortality.(3–5) The total projected annual number of deaths in men and women in India, attributable to tobacco use, is 1 million in the 2010s.(6) Representative information on tobacco use in India is available through several large-scale surveys.(7–10) However, these surveys often don’t refer to the quantity of product being consumed and the age of initiation. Epidemiological studies of coronary heart disease and its risk factors have either focused on rural areas(11–14) or on urban areas.(15–18) Kumar *et al*. studied the prevalence of tobacco use in villages, towns, and urban populations in northern India.(19) Previously we had reported a high prevalence of tobacco use among slum dwellers.(20) Overall, there is dearth of information on tobacco use especially in the urban-slum section of the Indian population.

The urban-slum population has emerged as a new section which is known to fare very poorly on issues related to health.(21) The proportion of the urban-slum population is also increasing at a rapid rate. In India, 28% of the total population was living in urban areas in 2001, with a future projection of about 38% (535 million) by 2026.(22) The National Sample Survey (NSS) 58th round reported that in India, 1 in 7 urban residents is a slum dweller.(23) Not much scientific literature exists on the patterns of tobacco use across the urban-rural and urban-slum populations. This information is required so as to enable development and implementation of effective intervention strategies. The objective of the present report is to analyze in detail the pattern of tobacco use in a north Indian community and its association with the residential strata, namely, urban, urban-slum, and rural.

## Materials and Methods

The analysis is based on data collected from a crosssectional survey of non-communicable disease risk factors which was carried out in the Ballabgarh block of Haryana, India, in years 2003-2004. The survey itself was a part of a multicentric survey by the Indian Council of Medical Research (ICMR) and World Health Organization (WHO). The study was conducting using the STEPwise approach to surveillance (STEPS) of NCD risk factors.(24)

The study was conducted at the Comprehensive Rural Health Services Project (CRHSP), Ballabgarh. The project is managed by All India Institute of Medical Sciences, New Delhi. Ballabgarh block includes the town of Ballabgarh and the nearby villages. Further, the district has industries which provide employment to a large number of labourers in the region. The population is a mix of urban, urban-slums, and rural.

Five age groups of 10 years each (15-64) were covered for both sexes in all three residence strata, in line with the recommendation of the STEPs manual.(24) The survey aimed at recruiting a minimum of 250 participants in each age-sex-residence stratum. Using a multistage sampling approach, the villages, slums, and colonies (within the town) were selected for the recruitment of participants. The houses for the selection of participants were selected based on a systematic random sampling approach in urban colonies. All the households in selected slums and the selected villages were approached. One male and one female were selected in alternate households after preparing a list of eligible individuals and selecting one randomly on the basis of the last digit of a currency note. Additional colonies, slums, and villages had to be sampled as there was a shortfall of participants in the younger and the eldest age groups. Revisits were made for locked houses at a different time of the day than the previous visit. The response rate of the study was extremely good at 98.1%. A total of 7981 individuals were interviewed during the study.

The survey instrument was a Hindi-translated, pretested version of the STEPs questionnaire administered by trained male and female field workers. The training was performed by the AIIMS-ICMR team of experts at CRHSP, Ballabgarh. Quality assurance was maintained by way of regular supervision by investigators and ICMR team. Data were collected on parameters related to tobacco use, alcoholism, diet, physical activity, blood pressure, waist circumference, body mass index, and diabetes mellitus. The definitions used for various parameters were as per the WHO STEPS guidelines.(20,24–26) Current daily smokers were defined as those who were currently smoking tobacco daily in the form of cigarettes, bidis (hand-manufactured cigarettes consisting of tobacco wrapped in a temburini leaf), hookah (Indian water pipe), chillum, or any other smoked form. Similarly, current daily smokeless tobacco users were defined as those who were currently using chewable tobacco products: khaini (tobacco-lime mixtures), gutkha (tobacco with betel nut, catechu, lime, and flavorings), naswar (snuff), or zarda paan (betel quid with tobacco) daily. Information was gathered on the age of initiation of smoking and the self-reported quantity of specific tobacco products consumed by the current daily users.

The computer-based entry was done by a data entry operator in FoxBASE. Ten per cent of the data were reentered by a second data entry operator and this was validated against the original entries. Weights were calculated of the various age-sex-residence groups against the WHO standard population.(27) Further details of methodology as well as age-adjusted prevalence (adjusted to local district population) have already been reported elsewhere.(20,25,26) The analysis was done using the SPSS 12.0 statistical package (SPSS Inc., Chicago, IL, USA).

The study was approved by the AIIMS from the ethical point of view. Written informed consents were taken from all the respondents beforehand. Any of the respondents requiring clinical management were referred to the non-communicable disease clinic running at CRHSP, Ballabgarh.

## Results

The target sample size of 250 was met in all the age-sex-residence groups. The educational attainment levels were higher for men than for women [Table 1]. Within the regions, the educational attainment was highest among the respondents in urban areas followed by urban-slums and the rural areas. The vast majority of the women were not engaged in any sort of occupation except for doing housework. Nearly a fifth of the rural respondents reported unemployment. Urban men tended to be occupied in white collar jobs and businesses. Education when analyzed separately for individuals under 25 years of age showed that educational attainment was much better with over 40% of men in all three regions still studying (data not shown).

**Table 1:** Distribution of respondents across age-gender-residence strata

Gender	Male			Female		
Site	Urban	Urban-slums	Rural	Urban	Urban-slums	Rural
Total number of respondents	1263	1260	1359	1326	1304	1469
Highest level of education among respondents aged 25-64 years						
No schooling/less than primary	6.3%	17.6%	25.1%	26.6%	58.4%	75.5%
Completed primary school	6.8%	14.1%	20.2%	13.8%	13.5%	11.4%
Secondary school completed	9.0%	16.0%	17.0%	12.0%	8.7%	7.4%
Senior secondary school and above	77.9%	52.3%	37.7%	47.6%	19.4%	5.7%
Main occupation among respondents aged 25-64 years						
Business/clerical	54.6%	40.3%	21.9%	9.3%	2.9%	1.8%
Labor/small business	31.4 %	42.4%	50.2%	1.9%	2.8%	0.6%
Students	0.8 %	0.3%	0.1%	0.2%	0.3%	0.2%
Homemaker	0%	0%	0%	87.8%	93.4%	97.1%
Unemployed/retired	13.1%	17.0%	27.8%	0.8%	0.7%	0.3%

In absolute terms, the proportion using tobacco was nearly 4% higher in rural men as compared to urban-slum men and 17% higher in rural men as compared to urban men [Table 2]. A similar pattern was seen for smoked tobacco use. On the other hand, for smokeless tobacco use, the pattern was reversed, with a proportionate use among urban men being nearly twice as common as in rural men. Among women, the overall tobacco use and current daily smoked tobacco use showed a similar pattern as in the men, while smokeless tobacco use was maximum in urban-slums.

**Table 2:** Tobacco use in the ballabgarh population (adjusted to the WHO standard population)

Gender	Male			Female		
Site	Urban (*n*=1263)	Urban-slums (*n*=1260)	Rural (*n*=1359)	Urban (*n*=1326)	Urban-slums (*n*=1304)	Rural (*n*=1469)
Daily current tobacco use	35.2%^a^	48.3%	52.6%	3.5%^a^	11.9%	17.7%
	(32.6- 37.9)	(45.5-51.0)	(49.9-55.3)	(2.6-4.6)	(10.2-13.8)	(15.8-19.7)
Daily smoking	25%^a^	40.8 %	47.9%	2.2%^a^	9.1%	16.5%
	(22.6- 27.5)	(38.1-43.6)	(45.2-50.6)	(1.5-3.1)	(7.6-10.8)	(14.6-18.5)
Daily smokeless tobacco user	12%^a^	10.5%	6.8%	1.4%^a^	3%	1.4%
	(10.3-14.0)	(8.8-12.3)	(5.5-8.2)	(0.8-2.1)	(2.1-4.1)	(0.9-2.2)
Simultaneous smoked and smokeless tobacco use	1.9%^a^	3.0%	2.1%	0	0.2%	0.2%
	(1.2-2.8)	(2.1-4.0)	(1.4-3.0)			
Mean number of cigarettes smoked by current daily smokers	6.0^a^	4.2	3.1	-b	-b	-b
	(4.8-7.2)	(2.9-5.4)	(2.2-4.0)			
Mean number of bidis smoked by current daily smokers	13.4	14.2	15.1	6.3	7.3	7.4
	(12.2-14.6)	(13.4-15.1)	(14.3-15.9)	(3.4-9.3)	(5.8-8.8)	(6.2-8.7)
Mean number of times, hookah/pipe/chillum smoked by current daily smokers	-b	3.1	2.9	-b	2.6	3.5
		(2.3-4.0)	(2.6-3.3)		(1.9-3.3)	(3.2-3.8)
Mean number of times gutkha consumed in a day by current daily smokeless tobacco users	4.3	3.6	4.5	-b	-b	-b
	(3.2-5.4)	(2.9-4.3)	(0.7-9.6)			
Mean number of times khaini consumed in a day by current daily smokeless tobacco users	6.5^a^	4.4	4.6	4.0	5.5	4.6
	(5.4-7.5)	(3.6-5.2)	(3.8-5.5)	(2.1-5.8)	(4.1-6.9)	(2.5-6.7)

The age distribution of the users was standardized against the WHO standard population. Values are shown as percent (95% confidence intervals) for proportions and mean (95% confidence intervals) for quantitative variables.

a Refers to the statistically significant *P* value of <0.05 by the χ^2^ test (for proportions) or one-way ANOVA/Welsh test (for means) across the three regions within a specific gender.

b Means were not calculated since the number of observations was less than 25.

**Figure 1:**
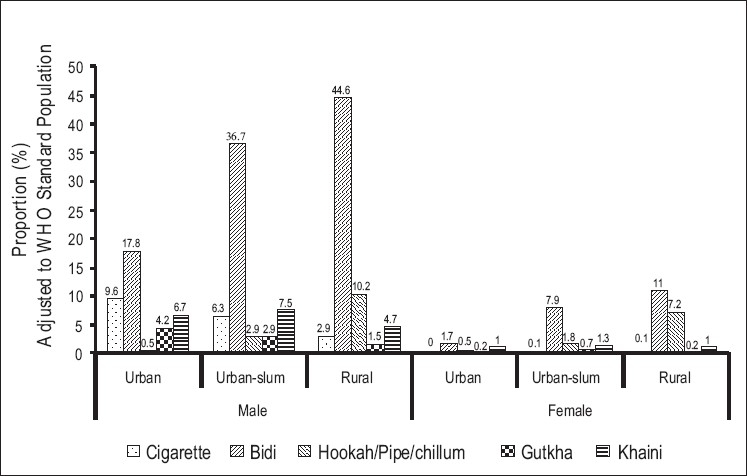
Consumption of specific tobacco products across residence and sex

The tobacco product which was most frequently used was bidi both in men and women [Figure 1]. Compared to urban males (17.8%), the proportion smoking bidi daily was double in urban-slums (36.7%) and triple in rural areas (44.6%). The use of cigarettes was lower than that of bidi in all three regions. While the use of bidis was highest in rural areas, the use of cigarettes was highest among urban respondents. In rural areas, hookah/pipe/chillum use turned out to be the second most preferred product after bidi, both among men and women. Among smokeless tobacco products, the most commonly used product was *khaini* followed by gutkha, in all three regions. The mean number of cigarettes smoked per day was double in urban compared to rural men. The mean number of bidis consumed per day by men was similar across all three regions. The mean age of smoking initiation among young smokers (15-34 year age group) was similar for all the three areas (urban 19.0 years, urban-slum 19.1 years, rural 18.7 years). The mean age of initiation of smoking among daily current smokers was higher in the older age groups (25.7 years in urban, 23.1 years in urban-slum, and 23.8 years in rural men in the 55-64 year age group) indicating a downward shift in the age of initiation in all three regions (data not shown in tables). The proportion of tobacco use increased consistently with the increasing age groups reaching a peak of 71.8% among 55- to 64-year-old rural men and 42.7% among 55- to 64-year-old rural women. The highest of daily smoking was seen among 45- to 64-year-old respondents whereas the highest daily smokeless tobacco use was seen among 25- to 34-year-old respondents. To study the relationship of current tobacco use with residence and education after adjusting for the effect of age, gender, and occupation, a logistic regression analysis was performed [Table 3]. All indicators of tobacco use (except cigarette use) were significantly associated with the site of residence. Further, we observed a significant association between lack of schooling and all forms of tobacco use except cigarettes.

**Table 3:** Association between the site of residence and educational status and tobacco use

Gender	Site of residence (Reference=urban)		Educational status (Reference=higher secondary school and above)		
	Rural	Urban-slum	No schooling/less than primary	Completed primary school	Secondary school completed
Current daily tobacco use	2	1.7	5.8	2.3	1.7
	(1.7-2.3)^a^	(1.4-2.0)^a^	(4.8-7.1)^a^	(1.9-2.8)^a^	(1.4-2.1)^a^
Daily smoking	2.3	2	4.4	2	1.7
	(2.5-3.5)^a^	(1.7-2.4)^a^	(3.6-5.4)^a^	(1.7-2.5)^a^	(1.4-2.1)^a^
Cigarettes use	0.4	0.8	0.3	0.5	0.6
	(0.3-0.6)^a^	(0.6-1.0)	(0.2-0.7)^a^	(0.3-0.8)^a^	(0.4-0.9)^a^
Bidi use	3.2	2.5	4.4	2.4	2
	(2.6-3.8)^a^	(2.1-3.0)^a^	(3.6-5.3)^a^	(2.0-3.0)^a^	(1.6-2.4)^a^
Hookah/pipe/chillum use	15.9	4	3	1.8	1.2
	(8.8-28.9)^a^	(2.1-7.6)^a^	(2.0-4.4)^a^	(1.2-2.9)^a^	(0.7-1.9)
Daily smokeless tobacco use	0.4	0.8	2.8	1.7	1.3
	(0.3-0.6)^a^	(0.7-1.1)	(2.1-3.6)^a^	(1.2-2.3)^a^	(1.0-1.8)
Gutkha use	0.3	0.7	1.8	1.7	0.8
	(0.2-0.5)^a^	(0.5-1.1)	(1.1-3.0)^a^	(1.0-2.8)	(0.4-1.5)
Khaini use	0.6	1	3.2	1.7	1.8
	(0.4-0.8)^a^	(0.7-1.3)	(2.3-4.5)^a^	(1.2-2.6)^a^	(1.2-2.5)^a^

Figures represent the odds ratio (95% confidence intervals) based on logistic regression analysis with age (years), gender, and occupation as covariates. While calculating odds ratio for association of educational status with tobacco use, analysis was restricted to 25- to 64-year-old respondents.

a Represents statistically significant results with a *P* value of less than 0.05.

## Discussion

The levels of tobacco use are high at all three strata. We have also observed a significant rural-urban-slum-urban gradient for tobacco use among men as well as women. There are different, and opposing, trends for use of smoked tobacco (more in rural areas) and smokeless tobacco (more in urban area) among men. Among women, the consumption of smokeless tobacco does not vary significantly across the three areas. The trends persist even after adjusting for the effect of potential confounders such as age, gender, literacy status, and occupation of the respondents. To the best of our knowledge, no other community-based study in India has systematically focused on all the three types of residential areas, namely, rural, urban-slum, and urban simultaneously while studying the spatial trend in tobacco consumption. We have adopted a standardized approach to the issue. The results are likely to have a high internal validity with a sufficient number of respondents to study inter-regional differences. Our results should not be interpreted to mean prevalence estimates for Ballabgarh since the results have been standardized against the WHO standard population. The point estimates might not be truly representative of the other northern states of India.

There is a gap in knowledge that exists on the association use of tobacco use with urban-slum residence. Kumar *et al*.’s study comes closest in terms of similarity of the population studied. However, they have not provided the exact definitions used for smoking and the data collection study was started over 10 years back. During this period, the definition of smoking too has undergone revisions. And it is very likely that the population characteristics too would have changed.(19) Reddy *et al*. studied non-communicable disease risk factors in industrial populations in highly urban, urban, and periurban areas in India and have come out with findings of much higher prevalence of tobacco use in periurban areas.(28) NSSO 62nd round (2005-2006) also reports that the proportion of the total household expenditure on tobacco and related products in rural households in India is triple of the consumption in urban households.(29) The recently conducted NFHS-3 reports that the proportion of male respondents consuming any form of tobacco is 49.9% in urban men and 61.1% in rural men. The prevalence of smoking as reported by the NFHS-3 is higher for rural as compared to urban regions.(10) The NFHS-3 was conducted in urban-slum populations as well but desegregated data for urban-slums are not yet available.

The use of smoked tobacco by urban-slum respondents is nearer to rural respondents (absolute difference of 4.3%) than the urban respondents (absolute difference of 15.8%). On the other hand, for smokeless forms of tobacco, the urban-slum respondents’ prevalence is much higher than that of the rural respondents (absolute difference of 3.7%) being nearer to the prevalence in urban populations (absolute difference of 1.5%). Simultaneous smoked and smokeless tobacco use was most commonly seen among the urban-slum respondents. The slum population mostly consists of recent migrants from the rural areas and from what we observe, they seem to be rapidly taking up the urban habits while still maintaining their rural habits. The risk of the development of certain disorders such as cancer of the oral cavity is known to be particularly high with the use of smokeless tobacco products. Thus the slum population becomes a highrisk group for the development of diseases associated both with smoked and smokeless forms of tobacco. The burden imposed by these disorders has the potential to further aggravate the already poor health status of these populations.

The most common form of tobacco being consumed in all the three populations in our study is *bidi*. The high prevalence of bidi use and smokeless tobacco use is seen among the rural and urban-slum respondents. The mean number of bidis smoked too was highest in these populations. These population groups also have a higher proportion of respondents who are poorly educated and are unemployed or engaged in lower paying jobs. These findings are supported by observations of Jindal *et al*. and Chaudhary *et al*. in rural as well as urban settings and by Chhabra *et al*. and Gupta *et al*. in urban settings.(30–33) Narayan *et al*. however had reported a higher proportion cigarette use compared to bidi use in urban population of Delhi.(34) A very likely reason for this observation is the pricing strategy of these tobacco products. Bidis cost nearly one-tenth of the cost of cigarettes. Gutkha and khaini, the two common smokeless tobacco products, are available for as little as half a rupee (approximately $0.01).

The prevalence of hookah use is high in the villages where it is the second most common form of tobacco used. The use of hookah is almost equally common among rural men and women. Hookah smoking is a habit that has been associated with Indian villages for several centuries. Over the years, the use of bidis and hookah has come to be very much ingrained in the rural villages of north India. It has become customary to offer bidis or hookah to visitors. Their ease of availability in the rural households is a likely reason for their predominance seen among rural women. The proportion of smokeless tobacco use is lower than smoking among women in all three regions. The pattern of tobacco consumption in women is known to show regional variations and the same could be the reason for our current observations. Our results also lend support to the general consensus that the mean age of initiation of smoking has been coming down. However, this result is subject to recall bias particularly for older age groups.(35) The observation of a high odds ratio of current daily tobacco use among illiterate men compared with higher secondary school educated males is supported by results published by the NFHS-3, Narayan *et al*., Gupta *et al*., and Subramanian*et al*.(10,34,36,37) The pattern that we observe is similar for all types of tobacco use except for cigarettes, where the odds of being a current cigarette smoker are higher among higher educated categories (even though this result is not significant in our study).

India is a signatory to the Framework Convention on Tobacco Control (FCTC) since September 2003. The Indian parliament has passed “The Cigarettes and Other Tobacco Products (Prohibition of Advertisement and Regulation of Trade and Commerce, Production, Supply and Distribution) Act” in 2003 (CTPA) and it was enforced from May 1, 2004. This Act calls for a ban on the sale of tobacco products to minors, a ban on direct and surrogate tobacco advertising (except at point of sale and on tobacco packs), and prohibition of smoking at public places. Even though the Indian Act is an important landmark step forward, there are still a few missing issues that need to be addressed as per the FCTC. Further, the implementation of the act has been lax as has been noted by Sinha *et al*. who found high levels of exposure to tobacco advertising by billboards among adolescents.(38) Further, there have been reports of use of pan masala (mixture or areca nut, slaked lime, catechu, and condiments) advertisements as surrogates for smokeless tobacco products especially gutkha.(39) Irregularities in the implementation of point-of-sale tobacco advertisements have also been reported.(40) A recent monograph notes that bidis are widely available in the remotest of Indian villages, branding is such that they strike a chord with the masses, and because the bidi-manufacturing industry is viewed as small scale, the current policies also seem to favor the bidi industry.(41) The issue of hookah smoking also does not figure in the current policies.

In light of the existing evidence and the results of the present study, it is clear that the tobacco control policy in India is not geared adequately toward addressing the issue and there is a need to modify the CTPA and widen its ambit. What our study stresses is the need to have rural orientation in the National Tobacco Control Programme that is currently being developed by the Government of India. There is also a need to focus on urban-slums as they are emerging as high-risk groups having a high prevalence of tobacco use. Our study reinforces the notion that bidis are the main tobacco product being consumed and as such the existing policies which favor the bidi industry need revision. Since the poorly educated individuals living in urbanslums and rural areas are at the maximum risk of using tobacco, identifying ways and means of reaching out to these communities will be critical to the success or failure of the program.
